# Validation of a Methodology for the Detection of Severe Acute Respiratory Syndrome Coronavirus 2 in Saliva by Real-Time Reverse Transcriptase-PCR

**DOI:** 10.3389/fpubh.2021.743300

**Published:** 2021-12-02

**Authors:** Daniel F. Escobar, Pablo Díaz, Diego Díaz-Dinamarca, Rodrigo Puentes, Pedro Alarcón, Bárbara Alarcón, Iván Rodríguez, Ricardo A. Manzo, Daniel A. Soto, Liliana Lamperti, Janepsy Díaz, Heriberto E. García-Escorza, Abel E. Vasquez

**Affiliations:** ^1^Sección de Biotecnología, Departamento Agencia Nacional de Dispositivos Médicos, Innovación y Desarrollo, Instituto de Salud Pública de Chile, Santiago, Chile; ^2^Departamento Agencia Nacional de Dispositivos Médicos, Innovación y Desarrollo, Instituto de Salud Pública de Chile, Santiago, Chile; ^3^Laboratorio de Biología Molecular, Hospital Guillermo Grant Benavente, Concepción, Chile; ^4^Laboratorio de Diagnóstico Molecular y Proteómica OMICs, Universidad de Concepción, Concepción, Chile; ^5^Dirección, Instituto de Salud Pública de Chile, Santiago, Chile; ^6^Departamento de Investigación, Postgrado y Educación Continua (DIPEC), Facultad de Ciencias de la Salud, Universidad del Alba, Santiago, Chile

**Keywords:** SARS-CoV-2, saliva, validation, RT-PCR, detection

## Abstract

In January 2021, the Chilean city of Concepción experienced a second wave of coronavirus 2019 (COVID-19) while in early April 2021, the entire country faced the same situation. This outbreak generated the need to modify and validate a method for detecting severe acute respiratory syndrome coronavirus 2 (SARS-CoV-2) in saliva, thereby expanding the capacity and versatility of testing for COVID-19. This study was conducted in February 2021 in the Chilean city of Concepción during which time, the town was under total quarantine. The study participants were mostly symptomatic (87.4%), not hospitalized, and attended care centers because of their health status rather than being asked by the researchers. People coming to the health center in Concepción to be tested for COVID-19 (*via* reverse transcriptase polymerase chain reaction [RT-PCR]) from a specimen of nasopharyngeal swab (NPS) were then invited to participate in this study. A total of 131 participants agreed to sign an informed consent and to provide saliva and NPS specimens to validate a method in terms of sensitivity, specificity, and statistical analysis of the cycle threshold (Ct) values from the RT-PCR. Calculations pertaining to the 127 participants who were ultimately included in the analysis showed sensitivity and specificity at 94.34% (95% CI: 84.34–98.82%) and 98.65% (95% CI: 92.70–99.97%), respectively. The saliva specimen showed a performance comparable to NPS as demonstrated by the diagnostic parameters. This RT-PCR method from the saliva specimen is a highly sensitive and specific alternative compared to the reference methodology, which uses the NPS specimen. This modified and validated method is intended for use in the *in vitro* diagnosis of SARS-CoV-2, which provides health authorities in Chile and local laboratories with a real testing alternative to RT-PCR from NPS.

## Introduction

Coronavirus disease 2019 (COVID-19), caused by severe acute respiratory syndrome CoV-2 (SARS-CoV-2), was first reported in December 2019 in Wuhan, China. The WHO subsequently declared it as a pandemic in March 2020 (1). Despite mitigation measures, the number of infected SARS-CoV-2 cases in Chile has increased dramatically since the beginning of the spread. From April 1, 2021 to April 14, 2021, a mean of 6,895 daily cases in Chile was detected, a positivity of 11.38%, and a mean of 93.8 daily deaths was reported (2). Given the national contingency, emergency vaccines have been approved for emergency use, focusing on health workers, chronically ill patients, and the elderly, reaching 4,815,079 people under the guidance of a complete vaccination scheme as of April 14 2021 (3).

The rapid spread of this disease is related to its highly infectious nature. It has been suggested that the disease is transmitted through saliva droplets and nasal discharge (4). The real-time reverse transcriptase polymerase chain reaction (RT-PCR) assay for SARS-CoV-2 using upper and lower respiratory tract specimens of the nasopharyngeal swab (NPS) is used as the reference technique for diagnosing COVID-19 (5). Although NPS is the most widely used biological specimen for the diagnosis of SARS-CoV-2, it has some limitations related to specimen collection and the safety of the healthcare workers. In this regard, the use of saliva samples for COVID-19 diagnosis and monitoring is emerging as a promising alternative to NPS for COVID-19. Saliva collection is a non-invasive method with the possibility of self-collection, thus circumventing the limitations associated with the use of NPS (6, 7).

Saliva has previously been shown to be useful for the detection of other respiratory viruses, such as influenza and human metapneumovirus and has comparable sensitivity/specificity in agreement with the NPS test (8). Regarding COVID-19 infection, *in vitro* analysis of RNA-sequencing (RNA-seq) profiles from four public and consensus datasets revealed the expression of the angiotensin converting enzyme 2 (ACE2) receptor in human granular cells in salivary glands (9). For this reason, saliva has been considered for use in the diagnosis of COVID-19 (10). The presence of SARS-CoV-2 in saliva could be related to different sources, such as viral entry into the oral cavity from the lower/upper respiratory tract (11) or by the release of viral particles into the oral cavity through the salivary ducts of infected salivary glands (12). We applied a cohort selection of cross-sectional study design (13) to Chilean study participants to validate a method using RT-PCR for RNA detection from SARS-CoV-2 virus in saliva specimens.

## Methods

### Study Design, Participants, and Sampling

The study obtained the authorization of the Scientific Ethics Committee of the Health Service of Concepción, Chile Number 20-01-02. Study subjects (men and women) between 11 and 77 years old who voluntarily agreed to sign an informed consent were included. Parents or legal guardians for volunteers under the age of 18 signed the informed consent. Patients were considered enrolled upon signing the informed consent.

A cohort-selection of cross-sectional study to assess diagnostic accuracy was used to validate a method using RT-PCR for genetically detecting the SARS-CoV-2 virus from saliva (candidate methodology herein refers to the use of raw saliva) and NPS as reference methodology specimens. This study was undertaken in the context of a performance evaluation of a Phase I *in vitro* diagnostic test as an exploratory manner. The objective was to obtain the first approximation of the diagnostic capacity of the new method (14) rather than making a field test using asymptomatic patients.

### Specimen Collection

Paired specimens of NPS and saliva were collected from symptomatic and asymptomatic individuals and from people who were in close contact with COVID-19 confirmed cases or who were clinically suspicious at the Multi-specialized Guillermo Grant Benavente Hospital (HGGB) and three Family Health Centers (FHCs) in the Chilean city of Concepción. First, the saliva specimen was obtained by means of self-sampling by the patient, who deposited a volume between 1 and 2 ml of saliva into a sterile nuclease-free 15 ml centrifuge tube. At least 30 min before sampling, each patient followed several specific guidelines: (1) no ingested food or liquids, except water; (2) no gum chewing; (3) no smoking or use of any type of mouth spray; and (4) no teeth brushing, no mouthwash, or dental floss. In addition, the participant had to remove cosmetic products, such as lip balms, and/or creams, when applicable. Once they provided the saliva specimen, the authorized healthcare workers performed the NPS specimen collection from each participant. All specimens were transported and maintained between 2 and 8°C until use within 24 h of collection after which they were stored at −20°C after the RNA isolation step (Supplementary Figure 1).

### Pretreatment of Saliva Specimens

The raw saliva and saliva samples diluted in a transport medium consisting of phosphate-buffered saline 1X (PBS-1X) were analyzed. This was done in order to determine if there is any statistical difference between the use of a raw saliva sample compared with a diluted one and compared against NPS. Raw saliva was diluted in the ratio of 1:1 by the addition of an equal volume of PBS-1X pH 7.4 without calcium/magnesium (Gibco by Thermo Fisher Scientific, MA, USA) into another tube and vortexed at full speed for 1 min and then both samples, the raw and diluted saliva, were centrifuged at 1,500 × g for 5 min to separate large debris (raw saliva and diluted saliva). Each specimen was labeled as “Saliva” or “Saliva + PBS.” The Saliva + PBS was included to evaluate the effect of dilution with this sample transport medium on the final results, both qualitatively and in terms of cycle threshold (Ct) values.

### Viral RNA Isolation

The RNA was isolated from a volume of 300 μl sample from the top fraction of each tube containing Saliva, Saliva + PBS, or NPS using the ExiPrep 96 Viral DNA/RNA Kit and the automated system ExiPrep™ 96 Lite (BIONEER, Daejeon, South Korea). Purified RNA was eluted in a final volume of 100 μl and used immediately or stored at −20°C until use.

### RT-PCR Procedure

All samples were screened using the TaqMan™ 2019-nCoV Assay Kit v1 (Applied Biosystems, CA, USA). RT-PCR assays and analysis were performed following the instructions of the manufacturer using the 7,500 Fast Real-Time PCR System thermocycler (Applied Biosystems, CA, USA). Briefly, the conditions of the thermal profile followed several specific steps: (1) 50°C for 5 min; (2) 95°C for 30 s; (3) 40 cycles at 95°C for 3 s, and (4) 60°C for 30 s. According to the instructions of the manufacturer in N-target, the Ct value <37 or two replicates with Ct values of 38 were considered positive; Ct values >39 were considered negative. An endogenous internal control (IC) was included in all reactions using primers and probe specifically directed at Human RnaseP-gene obtained from TaqMan^TM^ 2019-nCoV Assay Kit v1. Furthermore, positive, negative, and nontemplate controls were included in each run. According to the instructions of the manufacturer, IC at Ct value <40 was considered positive. A Ct value <30 in the positive control and no Ct values in negative and nontemplate controls were considered to validate runs.

### Quality Assurance of the Results

An IC was included in all the reactions using primers and a probe to Human RnaseP-gene was obtained from TaqManTM 2019-nCoV Assay Kit v1.

### Statistical Analysis

A 2 × 2 table was used in the assessment of diagnostic accuracy. Measures of diagnostic accuracy and the corresponding 95% of exact binomial CI (Clopper–Pearson intervals) were estimated (15). The Ct values were characterized using descriptive analyses, scatterplots, correlations, histograms, density charts, and P–P and boxplots. Also, CIs for the mean of the Ct values were estimated, and *t*-tests for paired data were used to compare Ct means between NPS, Saliva, and Saliva + PBS specimens. Bias and limits of agreement were estimated. A Bland–Altman plot was also constructed to assess the magnitude of disagreement (16). The data were analyzed with R (www.r-project.org) and R-Studio (www.rstudio.com).

## Results

**Figure 1:**
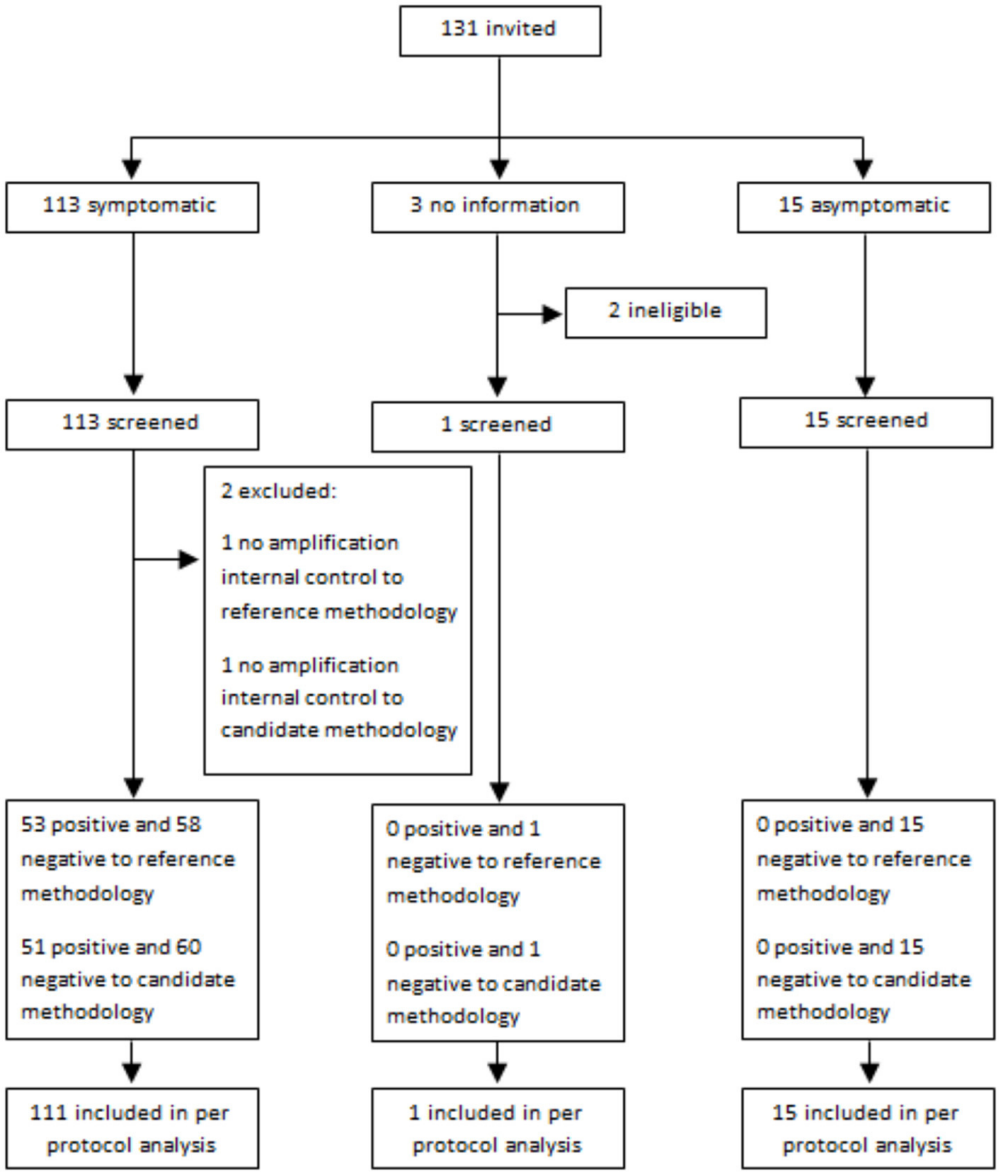
Flow of participants.

To analytically validate the use of saliva as a SARS-CoV-2 detection sample using RT-PCR, a total of 127 NPS and saliva samples were collected from the Chilean study participants (Table 1, Figure 1). Of these, 53 were positive and 74 were negative according to the RT-PCR results of the NPS samples, and 51 were positive and 76 were negative using the saliva sample (Supplementary Table 1).

**Table 1:** Demographic characteristics of the participants.

**Characteristics**	**n, (%)**
**Clinical aspects**	
Symptomatic	111 (87.4)
Asymptomatic	15 (11.8)
No information	1 (0.8)
**Motive**	
Close contact	21 (16.5)
Clinical suspicion	90 (70.9)
Travel	1 (0.8)
Other (ACF or no data available)	15 (11.8)
**Gender**	
Male	68 (53.5)
Female	59 (46.5)
**Age group**	
<18	5 (3.9)
18–34	69 (54.3)
35–50	29 (22.8)
51–65	20 (15.7)
>65	4 (3.1)
**Origin of specimens**	
Boca sur FHC	55 (43.3)
Lagunillas FHC	51 (40.2)
Víctor Manuel Fernández FHC	19 (14.9)
Guillermo Grant Benavente Hospital (Emergency Care Unit)	2 (1.6)

*ACF, active case finding; FHC, Family Health Centers*.

The performance of the Saliva method, with raw saliva was evaluated considering the following seven parameters: (1) sensitivity; (2) specificity; (3) positive predictive value (PPV); (4) negative predictive value (NPV); (5) positive likelihood ratio (PLR); (6) negative likelihood ratio (NLR); and (7) the Kappa value according to the criteria established by Landis and Koch (17) as shown in Table 2.

**Table 2:** Estimated diagnostic parameters with 95% CI.

**Parameter**	**Estimate**	**95% exact binominal CI (Clopper-Pearson intervals)**
Sensitivity	94.34%	(84.34–98.82%)
Specificity	98.65%	(92.70–99.97%)
PPV	98.94%	(89.55–99.95%)
NPV	96.05%	(88.89–99.18%)
LR+	69.81	–
LR–	0.06	-
Kappa	0.9349	(0.8721–0.9977)^a^

a *Estimated using the standard normal distribution. CI, Confidence Intervals; PPV, predictive positive value; NPV, negative predictive value; LR+, positive likelihood ratio; LR–, negative likelihood ratio. Parameters estimated from the results of Saliva compared with NPS*.

These data indicate that the saliva methodology detected 94.34% (95% CI: 84.34–98.82%) of the patients with COVID-19 and 98.65% (95% CI: 92.70–99.97%) of the patients without COVID-19. PPV and NPV values were 98.94% (95% CI: 89.55–99.95%) and 96.05% (95% CI: 88.89–99.18%), respectively (Table 2). The population prevalence of COVID-19 was not considered for the PPV and NPV estimations in this study. PLR was estimated at 69.81, and Cohen's kappa coefficient was estimated to measure the agreement between categorical variables. Contrary to a simple agreement of percentage between categories, this statistic considers the agreement that would be expected by chance. The kappa value was estimated at 0.9349 (95% CI: 0.8721–0.9977), which can be interpreted as almost perfect agreement according to the criteria established by Landis and Koch (17).

Descriptive statistics were used to summarize Ct values from NPS, Saliva, and Saliva + PBS (Supplementary Table 2). The mean and median Ct values for NPS were 23.51 and 22.84, respectively, which were higher than the mean and median Ct values for both Saliva (29.20 and 29.26) and Saliva + PBS (29.61 and 29.68). Furthermore, the minimum Ct value was lower for NPS (12.98) compared to Saliva (19.60) and Saliva + PBS (19.84), but maximum Ct values were similar, resulting in a wider Ct range for NPS. Coefficients of skewness and kurtosis were calculated at near 0 for NPS, Saliva, and Saliva + PBS, which may suggest that the data distribution of Ct values is symmetrical and possibly corresponds to the normal distribution. To determine whether an empirical correlation between specimens existed, a bivariate analysis was performed between Ct values from NPS and Saliva specimens (Supplementary Figure 2); from this figure, a low correlation of 0.47 could be identified between NPS and Saliva (Supplementary Figure 2A) and between NPS and Saliva + PBS with a correlation of 0.51 (Supplementary Figure 2B). However, a high correlation of 0.94 could be observed between Saliva and Saliva + PBS (Supplementary Figure 2C).

**Figure 2:**
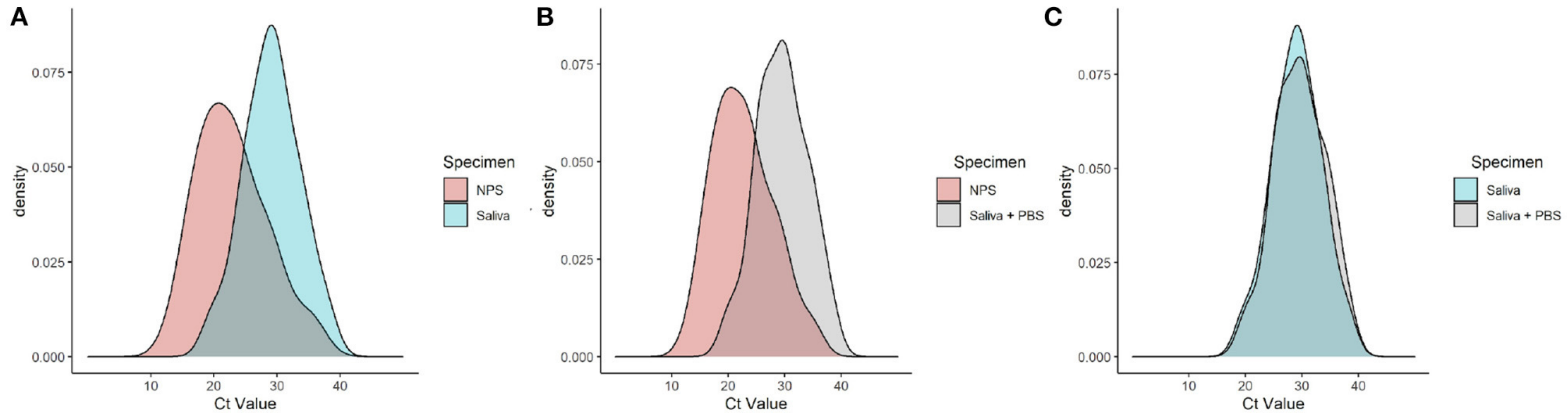
Density chart for cycle threshold (Ct) values from nasopharyngeal swab (NPS) and Saliva specimens **(A)**; NPS and Saliva + phosphate-buffered saline (PBS) specimens **(B)**; Saliva and Saliva + PBS specimens **(C)**. This plot compares Ct value distributions by means of smoothed density estimates (kernel density estimates).

**Figure 3:**
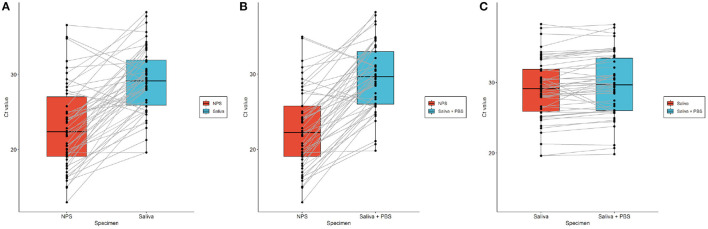
Boxplot for paired Ct values between NPS and Saliva **(A)**; NPS and Saliva + PBS **(B)**; and Saliva and Saliva + PBS **(C)**. Chart shows Ct values (black dots) obtained from NPS, Saliva and Saliva + PBS specimens paired with each other (gray lines) for each participant, along with boxplot and median marked as solid black line within each box.

Density charts from NPS, Saliva, and Saliva + PBS specimens are shown in Figure 2 as an illustrative approach to better understand the Ct value distribution. Higher Ct values were detected in Saliva and Saliva + PBS compared to NPS (Figures 3A,B), and there was no significant difference between the Ct comparison of Saliva and Saliva + PBS (Figure 3C). As previously mentioned, density characteristics are similar for NPS and Saliva specimens, suggesting that the probabilistic distribution of Ct values corresponds to the normal distribution.

To check the normality assumptions for the corresponding Ct values, P–P plots were constructed for each paired-positive dataset, considering that they are less sensitive to deviations in the tails and that differences in the middle are more detectable (Supplementary Figure 3). The Shapiro–Wilk test, which helps in the detection of a normal distribution, was used for each NPS, Saliva, and Saliva + PBS sample dataset with paired-positive matrix results (Supplementary Table 3). The points from the NPS datasets that are deviated from the theoretical diagonal line in the middle section, indicate a possible deviation from the normal distribution (Supplementary Figure 3). However, the Shapiro–Wilk test results indicate that with a 95% CI, the hypothesis stating that sample datasets coming from a population that has a normal distribution cannot be rejected. To assess the Ct value distribution and quartiles, a boxplot is shown in Supplementary Figure 4. In general, Ct values for Saliva and Saliva + PBS specimens are higher than those for NPS specimens. Also, the median, marked at the center of each box, indicates a symmetrical distribution, a finding that further supports the previous descriptive analysis.

Paired *t*-tests were used to determine whether there was a statistically significant difference in the mean of Ct values between NPS and Saliva specimens. The null hypothesis of or the mean difference between paired specimens was rejected for NPS vs. Saliva, NPS vs. Saliva + PBS, and Saliva vs. Saliva + PBS, considering a 95% CI. Although no mean difference was expected between Saliva and Saliva + PBS according to previous analyses, the paired *t*-test was significant. Conditioning this parametric test as a paired test that directly affected the estimated *p*-value, an independent *t*-test would have given a nonsignificant result (*t*: −0.6045; *p*-value: 0.547). Although Ct values between Saliva and Saliva + PBS seemed similar among previous analyses, paired specimens had a difference of over 10% in Ct value, which might explain the significant paired *t*-test result (Figure 3, Supplementary Table 4).

Descriptive statistics were used to summarize the difference in Ct values between NPS, Saliva, and Saliva + PBS (Supplementary Table 5). The range was lower in the difference between Saliva and Saliva + PBS compared to other differences, indicating that Ct values were similar. The mean and median along with the coefficient of skewness and kurtosis indicate a possible normal distribution for the difference between NPS and Saliva in addition to NPS and Saliva + PBS. Nevertheless, normal distribution characteristics were not observed for the difference between Saliva and Saliva + PBS for which the coefficients of skewness and kurtosis lie further from the referential value of 0.

Histogram and density charts for Ct value difference were analyzed to carry out an agreement analysis based on a comparison method proposed by Altman and Bland (16) (Supplementary Figure 5. A Gaussian-shaped distribution was seen for the difference between NPS and Saliva in addition to the difference between NPS and Saliva + PBS but slightly skewed to the right (Supplementary Figures 5A,B). A large deviation from the normal distribution can be seen for the difference between Saliva and Saliva + PBS, which was also mentioned in the descriptive analysis (Supplementary Figure 5C). P–P plots and the Shapiro–Wilk test were assessed to check normality assumptions for Ct value differences (Supplementary Figure 6, Supplementary Table 6). Density plots (Supplementary Figure 5C) suggest a large deviation from the normal distribution for the difference between Saliva and Saliva + PBS. Points deviate in both extremes and the middle section of the plot; they also transcend confidence bands. The Shapiro–Wilk test concurred with P-P plot interpretations. The results indicate statistical significance (W: 0.8102; *p*-value: 1.521^*^10^−6^) for Ct value difference between Saliva and Saliva + PBS. In other words, with a 95% CI, the null hypothesis, stating that the Ct value difference comes from a population with a normal distribution, was rejected. The median was toward the upper part of the box for the Ct value difference between NPS and Saliva and for NPS and Saliva + PBS, which suggests a slight deviation from normal distribution characteristics (Supplementary Figure 7). The Ct value difference between Saliva and Saliva + PBS was concentrated within a small range, and atypical data points were also identified (marked as atypical beyond calculated boxplot “whiskers”). This extreme value data might contribute to the non-normal distribution results mentioned from the P–P plot and Shapiro–Wilk test (Supplementary Figure 5C).

To estimate whether both the methods, NPS and Saliva were equivalent and, hence, interchangeable, an agreement analysis was performed. Since the differences between NPS and Saliva, and between NPS and Saliva + PBS proved to be normally distributed according to the previous analyses, agreement parameters and CIs were estimated following the method of statistical comparison proposed by Altman and Bland (18) as shown in Supplementary Figures 8, 9, Supplementary Tables 7, 8. For Bland–Altman plots, limits of agreement are estimated based on the assumption that the differences are normally distributed. Essentially, these plots do not indicate whether the agreement is suitable for one method or the other but rather quantifies the bias (estimated by the mean difference) and a range of agreement.

## Discussion

The COVID pandemic is an ongoing public health crisis. At the request of the Ministry of Health from Chile, our laboratory validated a new methodology for the detection of SARS-CoV-2. The use of saliva has several advantages compared to the collection of NPS, such as the reduction of contact with healthcare workers and the low use of consumables; also, it causes less discomfort to patients. This report validated the RT-PCR methodology from saliva, producing a sensitivity and specificity of 94.34 and 98.65%, respectively. This method provides an excellent alternative to an NPS SARS-CoV-2 detection. The Saliva method of detection is already being implemented in various regions of our country.

Previous reports, where the authors compared saliva and NPS to SARS-CoV-2 detection by RT-PCR, on the date of this study execution showed considerable differences between their results, finding highly variable sensitivity and specificity percentages in the range of 31–100% and 70.33–100%, respectively (7, 19–25). The candidate method (RT-PCR using saliva) validated in this study was performed in parallel with the reference methodology (RT-PCR using NPS) from the moment of specimen collection, which contributes important value to the comparison between the two. In addition, each specimen of NPS, Saliva, and Saliva + PBS was processed during a period lasting <24 h from collection.

Previously, it has been described that there are changes in the detection of the virus using Saliva + PBS and Saliva, for which the present study characterized the effect of the transport medium in the detection of SARS-CoV-2 employing the analysis of Ct by RT-PCR (26, 27). Diagnostic parameters were calculated based on 53 positive and 74 negative NPS specimens. Performance from the candidate methodology was evaluated with a sensitivity and specificity of 94.34% (95% CI: 84.34–98.82%) and 98.65% (95% CI: 92.70–99.97%), which is very similar to previous reports (24, 25, 28–31). The calculated PPV and NPV values imply that 1.96% of the positive saliva (raw) results were false positives and that 3.95% of negative saliva results were false negatives (Supplementary Tables 1, 2). The calculated PLR value implies that it is 70 times more likely for an infected patient (positive NPS patient) to obtain a positive saliva result than a patient who does not have the disease (negative NPS patient) to obtain the same result. Furthermore, NLR was estimated at 0.06, meaning that a negative saliva result is 0.06 times less likely in infected patients than in patients without the disease. In other words, a negative saliva result is 16.7 (1/0.06) times more likely in patients without the disease than in the infected patients (Table 2). Another way to interpret this result is to express that one false negative for approximately 17 true negative results will be found. Only one false positive and three false negatives were detected (Supplementary Table 1). In the case of the false positive, the replicates yielded Ct values in the range of 33.24–33.45 in Saliva and 34.22–34.64 in Saliva + PBS. A similar disagreement was previously reported; participants were tested positive for SARS-CoV-2 in Saliva and not in NPS (30). One of the limitations of this study was that the three participants who were negative for Saliva and positive for NPS could not be resampled. We hypothesized that the three specimens, having CT with a mean at 33.05 (31.41–36.05) in the NPS had a low viral load at the nasopharyngeal and undetectable viral load in the saliva level. However, the methods presented here had a high correlation.

Previous reports have described that the viral load of SARS-CoV-2 in saliva is lower than in the NPS (24, 25, 32). These reports suggest that a lower viral load is expected in saliva than in NPS even when studies that present opposite results have been reported in which the Ct values are lower than those obtained from NPS (33). In our study, comprising 127 participants, 53 (41.73%) were positive from the NPS specimen with a mean of 23.51 ± 5.93 for the Ct values, 51 (40.16%) were positive from saliva with a mean of 29.20 ± 4.39) for the Ct values, and 50 (39.37%) were positive in the Saliva + PBS group with a mean of 29.61 ± 4.44 for the Ct values. Despite the differences in Ct, the diagnostic capacity of our method allows us to identify people infected with the virus.

Furthermore, Ct values were analyzed regarding variation, distribution, and agreement characteristics among specimens. A descriptive summary of Ct values suggests that the mean and median values were lower for the Ct values of NPS rather than for Saliva or Saliva + PBS. The coefficients of skewness and kurtosis also suggest that the distribution of Ct values was symmetric for all three specimens. Sample datasets with paired-positive matrix results were analyzed using density charts and P-P plots to check for a hypothesized normal distribution. The Shapiro–Wilk test complemented the previous analyses, and significant results were obtained, indicating that each sample dataset is normally distributed with a 95% CI. Paired *t*-tests suggest statistically significant mean differences between NPS and Saliva (*t*: −8.367; *p*-value: 5.282^*^10^−11^), NPS and Saliva + PBS (*t*: −9.902; *p*-value: 3.495^*^10^−13^), and Saliva and Saliva + PBS (*t*: −2.564; *p*-value: 1.346^*^10^−2^). The significant results from the paired *t*-test between Saliva and Saliva + PBS might have been affected by four paired specimens with a Ct value difference >10%. The Bland–Altman agreement analysis was performed between NPS and Saliva and also between NPS and Saliva + PBS. This analysis was not carried out for the Ct value difference between Saliva and Saliva + PBS because it was neither considered as a part of the main objective of this article nor did it comply with normal distribution assumptions. The Bland–Altman plot showed biases of −6.18 and −6.85 units for the difference between NPS and Saliva, and NPS and Saliva + PBS, respectively, suggesting that Saliva Ct values were on average, 6.18 and 6.85 units >NPS Ct values. Bias was considered significant for both paired comparisons, a finding that agrees with significant paired *t*-test results. No trends or patterns were observed between Ct value difference and specimen means, which indicates that differences between paired specimens do not appear to have been affected by the magnitude of Ct values.

As previously mentioned, paired statistical analyses indicate differences in Ct values among NPS, Saliva, and Saliva + PBS. A statistically significant paired *t*-test also suggests that Ct values between relatively similar specimens (Saliva and Saliva + PBS) were different. However, four paired specimens might have affected the test sensitivity within the compared dataset. Agreement analysis between specimens also supports paired differences among Ct values. Although a quantitative approach used for method comparison strongly suggests differences in specimen Ct values, the qualitative analysis seems to indicate that diagnostic interpretations are highly similar between the candidate (saliva) and the reference method. Sensitivity and specificity diagnostic values were considered relevant to conclude that similar categorical test results can be obtained from both methodologies despite quantitative Ct value statistical differences between specimens.

It is important to be cautious and carry out a preliminary study to apply this methodology in the active search for cases of the asymptomatic population. Here, only 15 (11.8%) participants did not present symptoms related to COVID-19 at the time of sampling, all being negative in both NPS and Saliva. In addition, the date of the onset of symptoms in participants who did present symptoms associated with COVID-19 is not available in this study, which is also a limitation to be considered in future research.

On the other hand, using the saliva specimen has several advantages, among them; non-invasive compared to NPS, reduces the risk of transmission for the health personnel who take the NPS specimen, since the saliva collection is self-sampled by the patients. For self-sampling purposes, it is easier to obtain a reliable saliva specimen than a nasopharyngeal specimen; the easy collection allows for sampling in children and the elderly who are particularly anxious. It allows to carry out tests for the early detection of COVID-19 without causing physical discomfort to patients (34).

These findings allowed us to assert that the validated methodology for detecting SARS-CoV-2 in saliva presents a similar performance to the method from NPS, and healthcare centers can be used in patients who require a COVID-19 diagnosis. Furthermore, this work serves as a reference for implementing RT-PCR in saliva in the care centers of the public healthcare network at the suggestion of the Ministry of Health from Chile.

## Data Availability Statement

The original contributions presented in the study are included in the article/Supplementary Material, further inquiries can be directed to the corresponding author/s.

## Ethics Statement

The studies involving human participants were reviewed and approved by Scientific Ethics Committee of the Health Service of Concepción, Chile Number 20-01-02. The patients/participants provided their written informed consent to participate in this study.

## Author Contributions

DE, PD, PA, and BA performed analyses. RP, DE, PD, DD-D, RM, DS, and AV interpreted the data. DE, PD, RP, and DD-D wrote the first draft of the manuscript. AV, JD, DE, PD, RP, DD-D, LL, HG-E, and IR conceived and designed the study. AV, LL, IR, JD, HG-E, DE, DD-D, PD, RM, and DS made critical revisions to the manuscript. All authors have read and approved the final manuscript.

## Funding

This study was supported by Health Public Institute of Chile.

## Conflict of Interest

The authors declare that the research was conducted in the absence of any commercial or financial relationships that could be construed as a potential conflict of interest.

## Publisher's Note

All claims expressed in this article are solely those of the authors and do not necessarily represent those of their affiliated organizations, or those of the publisher, the editors and the reviewers. Any product that may be evaluated in this article, or claim that may be made by its manufacturer, is not guaranteed or endorsed by the publisher.
