# Supplementary material for: Validation of a Methodology for the Detection of Severe Acute Respiratory Syndrome Coronavirus 2 in Saliva by Real-Time Reverse Transcriptase-PCR
Source: Front Public Health. 2021 Dec 2;9:743300. doi: 10.3389/fpubh.2021.743300 (PMC8674452; doi:10.3389/fpubh.2021.743300)

**Supplementary Data**

**Supplementary Table 1.** Positive and negative results for severe acute respiratory syndrome coronavirus 2 (SARS-CoV-2) detection from nasopharyngeal swab (NPS) and Saliva samples.

|  |  | NPS | | |
| --- | --- | --- | --- | --- |
|  |  | Positive | Negative | Total |
| Saliva | Positive | 50 | 1 | 51 |
|  | Negative | 3 | 73 | 76 |
|  | Total | 53 | 74 | 127 |

**Supplementary Table 2.** Descriptive summary of cycle threshold (Ct) values by specimen.

| Specimen | n | Mean | SD | Median | Minimum | Maximum | Range | CS | CK |
| --- | --- | --- | --- | --- | --- | --- | --- | --- | --- |
| NPS | 53 | 23.51 | 5.93 | 22.84 | 12.98 | 36.58 | 23.6 | 0.44 | –0.70 |
| Saliva | 51 | 29.20 | 4.39 | 29.26 | 19.60 | 38.30 | 18.7 | –0.07 | –0.45 |
| Saliva + PBS | 50 | 29.61 | 4.44 | 29.68 | 19.84 | 38.24 | 18.4 | –0.06 | –0.65 |

Abbreviations: SD: Standard Deviation; CS: Coefficient of Skewness; CK: Coefficient of Kurtosis.

**Supplementary Table 3.** Shapiro–Wilk test for Ct values from each NPS, Saliva, and Saliva+ phosphate-buffered saline (PBS) specimen datasets with paired-positive specimen result. Note that six datasets were obtained considering that the dataset of one specimen was not the same throughout all paired-positive analyses; thus, three different paired combinations for which the specimen dataset differed after filtering both specimens to be positive were obtained (if for a certain pair, one specimen was positive and the other negative, or both negative, it would not constitute part of the paired-positive dataset).

| Dataset | W statistic | *p*-value |
| --- | --- | --- |
| NPS (paired-positive w/ Saliva) | 0.968 | 0.191 |
| NPS (paired-positive w/ Saliva+PBS) | 0.973 | 0.322 |
| Saliva (paired-positive w/ NPS) | 0.989 | 0.908 |
| Saliva (paired-positive w/ Saliva+PBS) | 0.988 | 0.900 |
| Saliva+PBS (paired-positive w/ NPS) | 0.980 | 0.570 |
| Saliva+PBS (paired-positive w/ Saliva+PBS) | 0.979 | 0.512 |

**Supplementary Table 4.** Paired *t*-tests for Ct values by matrix.

| Specimens tested | *t* statistic | *p*-value |
| --- | --- | --- |
| NPS vs Saliva | –8.367 | 5.282*10^-11^ |
| NPS vs Saliva+PBS | –9.902 | 3.495*10^-13^ |
| Saliva vs Saliva+PBS | –2.564 | 1.346-10^-2^ |

**Supplementary Table 5.** Descriptive summary for Ct value difference between NPS and Saliva; NPS and Saliva+PBS; Saliva and Saliva+PBS.

| Specimen Ct value difference | n | Mean | SD | Median | Minimum | Maximum | Range | CS | CK |
| --- | --- | --- | --- | --- | --- | --- | --- | --- | --- |
| NPS - Saliva | 50 | –6.18 | 5.23 | –6.30 | –15.79 | 5.77 | 21.56 | 0.33 | –0.40 |
| NPS - (Saliva+ PBS) | 49 | –6.85 | 4.84 | –6.48 | –16.36 | 3.92 | 20.27 | 0.40 | –0.27 |
| Saliva - (Saliva+ PBS) | 50 | –0.53 | 1.46 | –0.71 | –4.91 | 4.73 | 9.64 | 0.98 | 5.00 |

Abbreviations: SD: Standard Deviation; CS: Coefficient of Skewness; CK: Coefficient of Kurtosis.

**Supplementary Table 6.** Shapiro–Wilk test for Ct value difference between NPS and Saliva; NPS and Saliva+PBS; Saliva and Saliva+PBS.

| Specimen difference | W statistic | *p*-value |
| --- | --- | --- |
| NPS - Saliva | 0.976 | 3.999*10^-01^ |
| NPS - Saliva+PBS | 0.970 | 2.318*10^-01^ |
| Saliva - Saliva+PBS | 0.810 | 1.521*10^-06^ |

**Supplementary Table 7.** Bland–Altman parameters for Ct value comparison between NPS and Saliva.

| Parameter | Estimate | 95% CI | |
| --- | --- | --- | --- |
|  |  | Lower bound | Upper bound |
| Bias (n=50) | –6.18 | –7.67 | –4.70 |
| Lower limit of agreement | –16.43 | –18.98 | –13.87 |
| Upper limit of agreement | 4.06 | 1.50 | 6.61 |

Abbreviations: CI: Confidence Interval.

**Supplementary Table 8.** Bland–Altman parameters for Ct value comparison between NPS and Saliva+PBS.

| Parameter | Estimate | 95% CI | |
| --- | --- | --- | --- |
|  |  | Lower bound | Upper bound |
| Bias (n=49) | –6.85 | –8.24 | –5.46 |
| Lower limit of agreement | –16.34 | –18.73 | –13.94 |
| Upper limit of agreement | 2.64 | 0.25 | 5.03 |

Abbreviations: CI: Confidence Interval.

**Supplementary Figure Legends**

**Supplementary Figure 1.** Study Design. A schematic representation of the daily workflow performed by different participating institutions in this study. The scheme was repeated for six consecutive days. The Molecular Biology Laboratory facilities at Guillermo Grant Benavente Hospital and Molecular Diagnostic Laboratory and Proteomics OMICs at the University of Concepcion were used to carry out the preanalytical and analytical phases by the Health Public Institute´s authorized personnel. NPS: nasopharyngeal swab; RNA: ribonucleic acid; RT-PCR: real time reverse transcription polymerase chain reaction.

**Supplementary Figure 2**. Scatterplot matrix and correlation between NPS and Saliva (A); NPS and Saliva+PBS (B); and Saliva and Saliva+PBS (C).

**Supplementary Figure 3.** P–P plot with confidence bands based on a bootstrap at 95% confidence level for Ct values from six paired-positive datasets: NPS specimen dataset used for paired analysis with Saliva (A), Saliva specimen dataset used for paired analysis with NPS (B), NPS specimen dataset used for paired analysis with Saliva+PBS (C), Saliva+PBS specimen dataset used for paired analysis with NPS (D), Saliva specimen dataset used for paired analysis with Saliva+PBS (E), Saliva+PBS specimen dataset used for paired analysis with Saliva (F). The figure shows the cumulative probability points of Ct values (sample cumulative proportion) against the theoretical cumulative probability based on the normal distribution (theoretical cumulative proportion) for each Ct value. Note that six datasets were obtained considering that the dataset of one specimen was not the same throughout all paired-positive analyses; thus, three different paired combinations for which the specimen dataset differed after filtering both specimens to be positive were obtained (if for a certain pair, one specimen was positive and the other negative, or both negative, it would not constitute part of the paired-positive dataset).

**Supplementary Figure 4**. Boxplot for Ct values of both N-gene and Human RnaseP-gene from NPS, Saliva and Saliva+PBS specimens. Human RnaseP-gene was used as an internal control.

**Supplementary Figure 5.** Histogram and density chart for Ct value difference between NPS and Saliva (A); NPS and Saliva+PBS (B); Saliva and Saliva+PBS (C). The y-axis represents relative frequencies; hence, the histogram has a total area of one.

**Supplementary Figure 6.** P–P plot with confidence bands based on a bootstrap at 95% confidence level for Ct value difference between NPS and Saliva (A); NPS and Saliva+PBS (B); Saliva and Saliva+PBS (C). This figure plots the cumulative probability points of the Ct value differences (sample cumulative proportion) against the theoretical cumulative probability based on the normal distribution (theoretical cumulative proportion) for each Ct value difference. Note that 3 datasets were obtained considering the difference between paired-positive specimen datasets.

**Supplementary Figure 7.** Boxplot for Ct value difference between NPS and Saliva; NPS and Saliva+PBS; Saliva and Saliva+PBS.

**Supplementary Figure 8.** Bland–Altman plot with Bias, agreement limits and corresponding 95% confidence intervals for Ct value difference between NPS and Saliva.

**Supplementary Figure 9.** Bland–Altman plot with bias, agreement limits and corresponding 95% confidence intervals for Ct value differences between NPS and Saliva+PBS.

Supplementary figure 1.


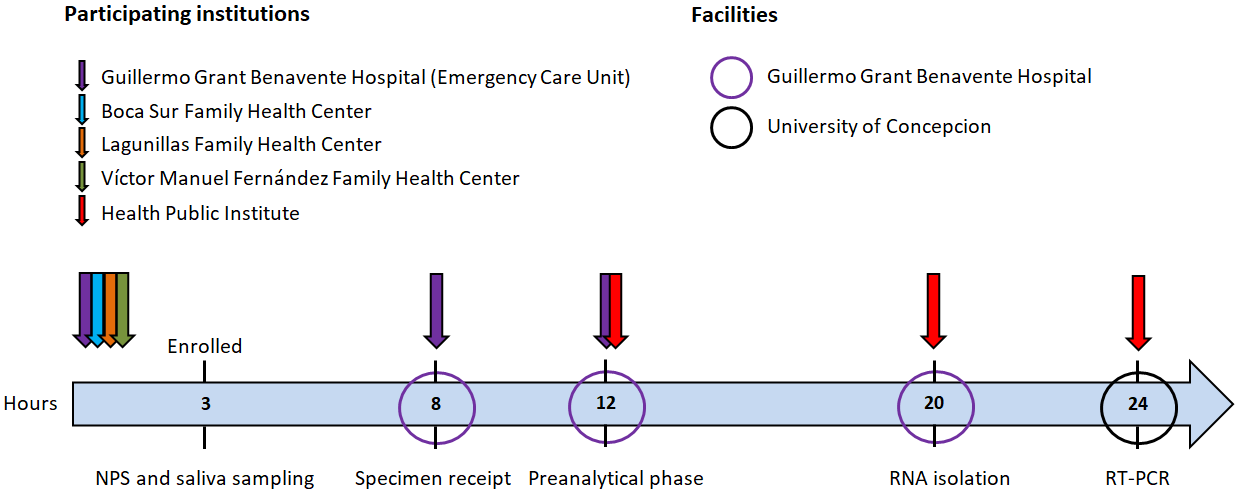


Supplementary figure 2.


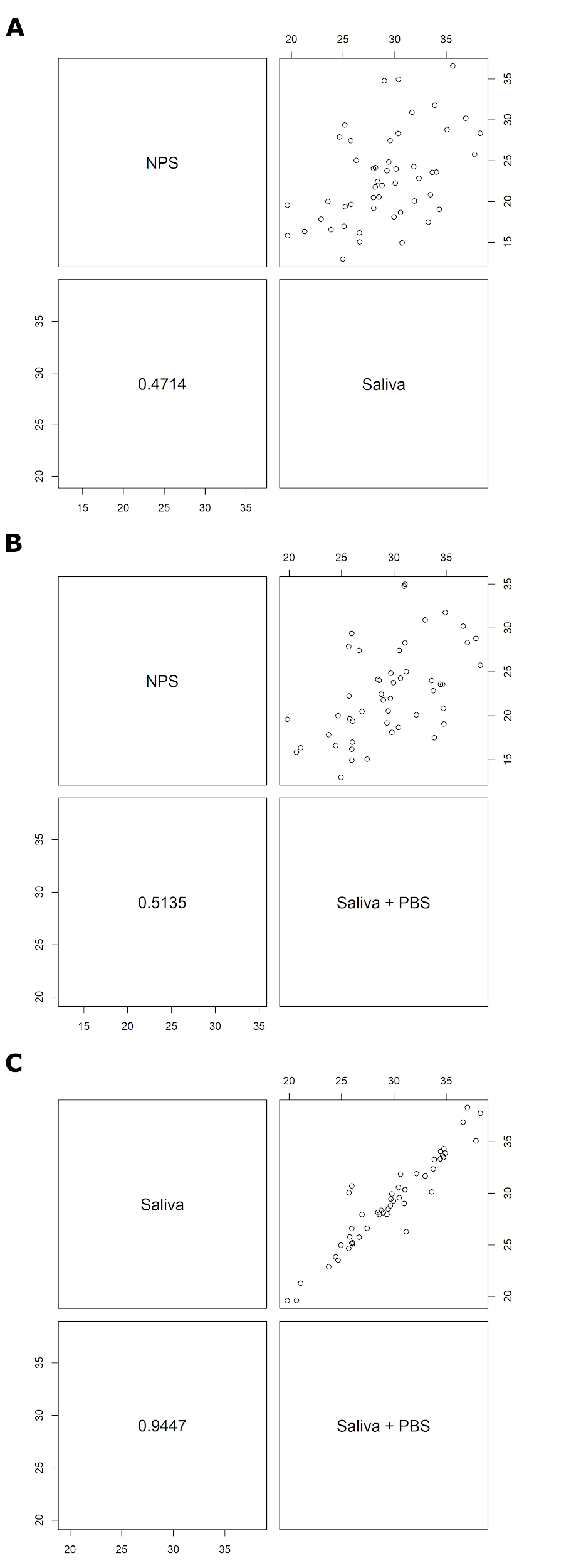


Supplementary figure 3.


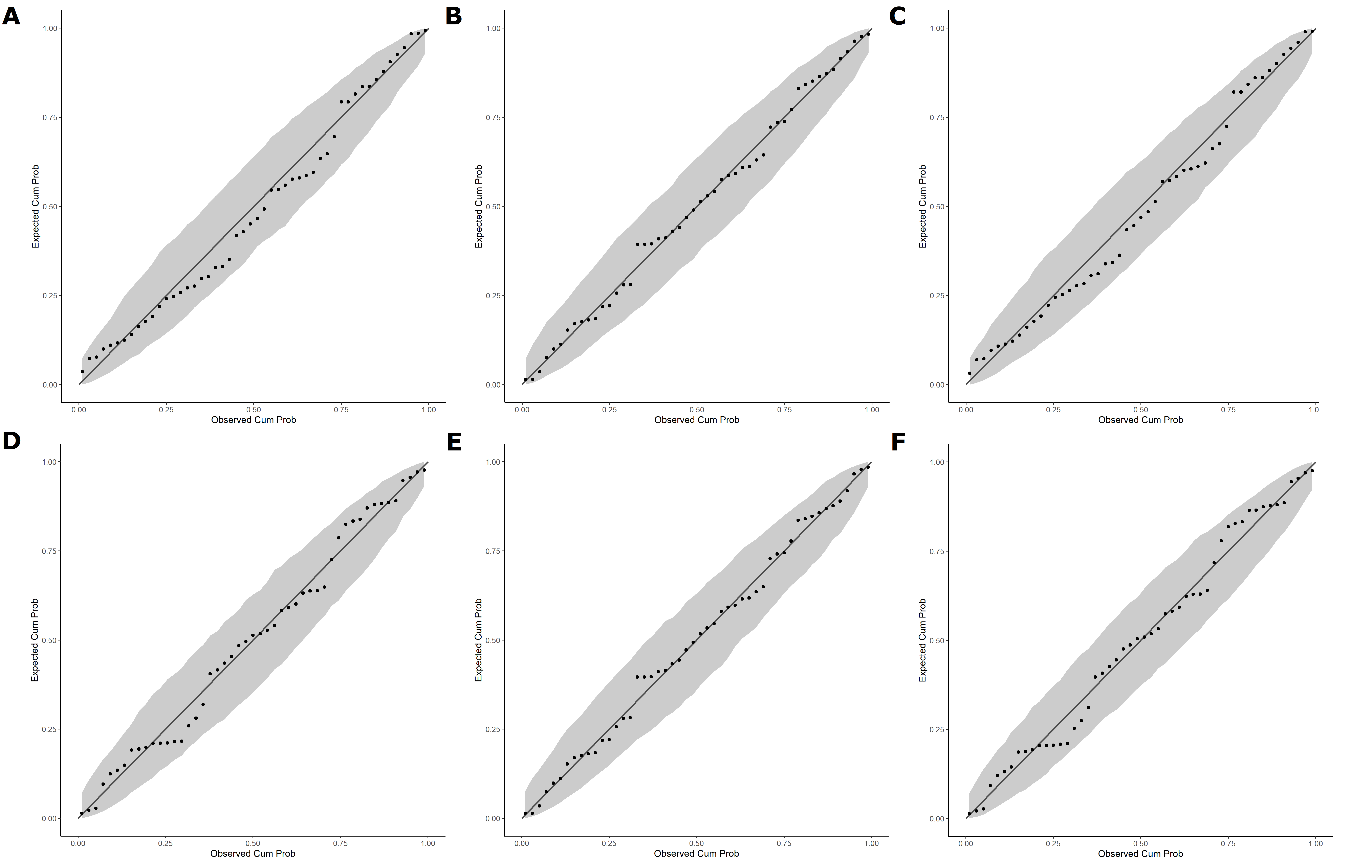


Supplementary figure 4.


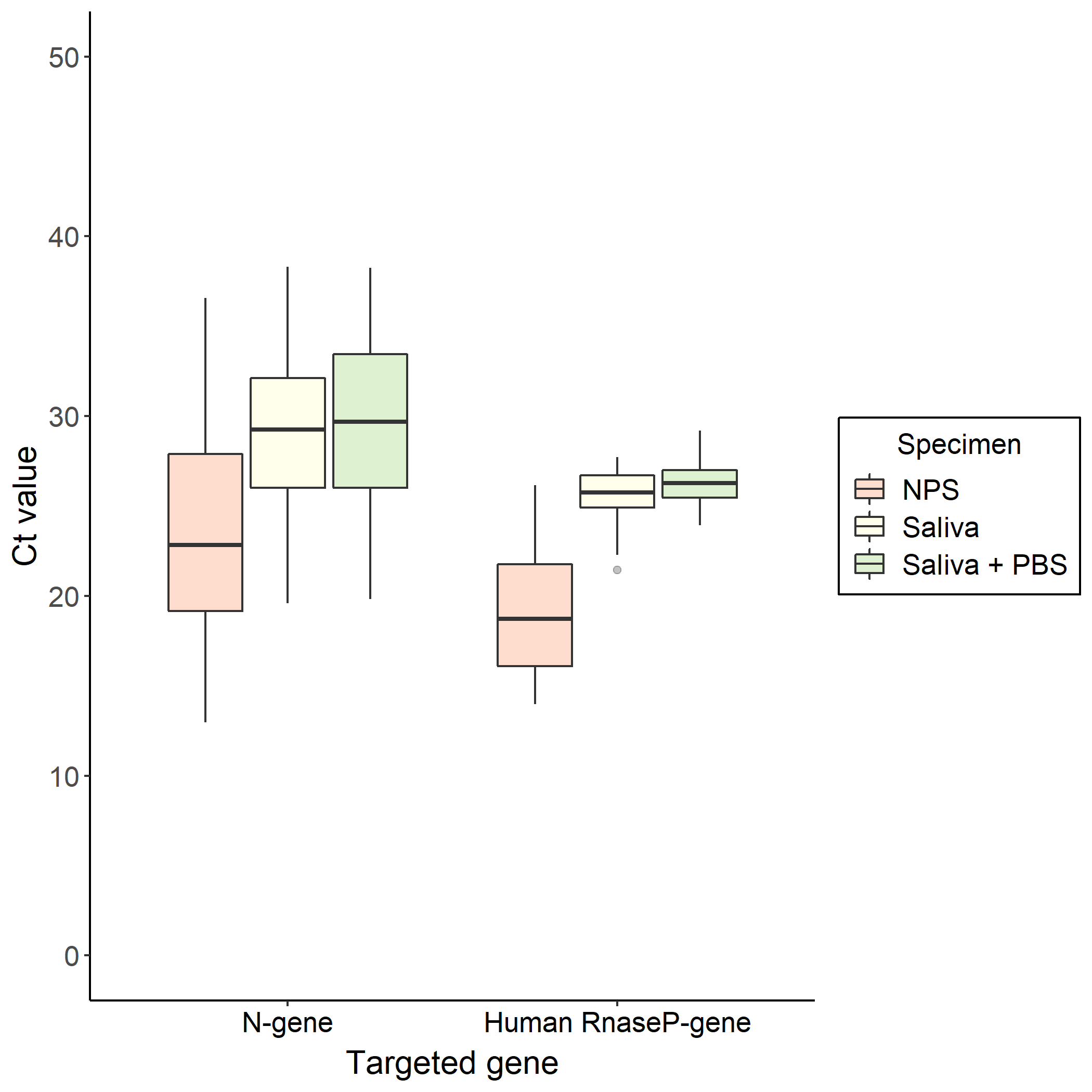


Supplementary figure 5.


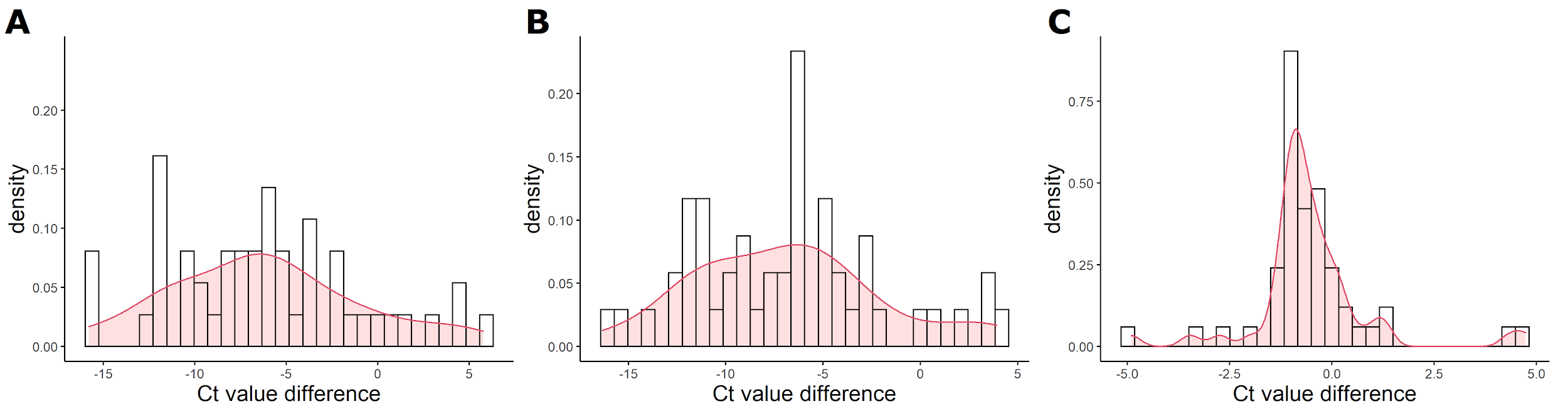


Supplementary figure 6.


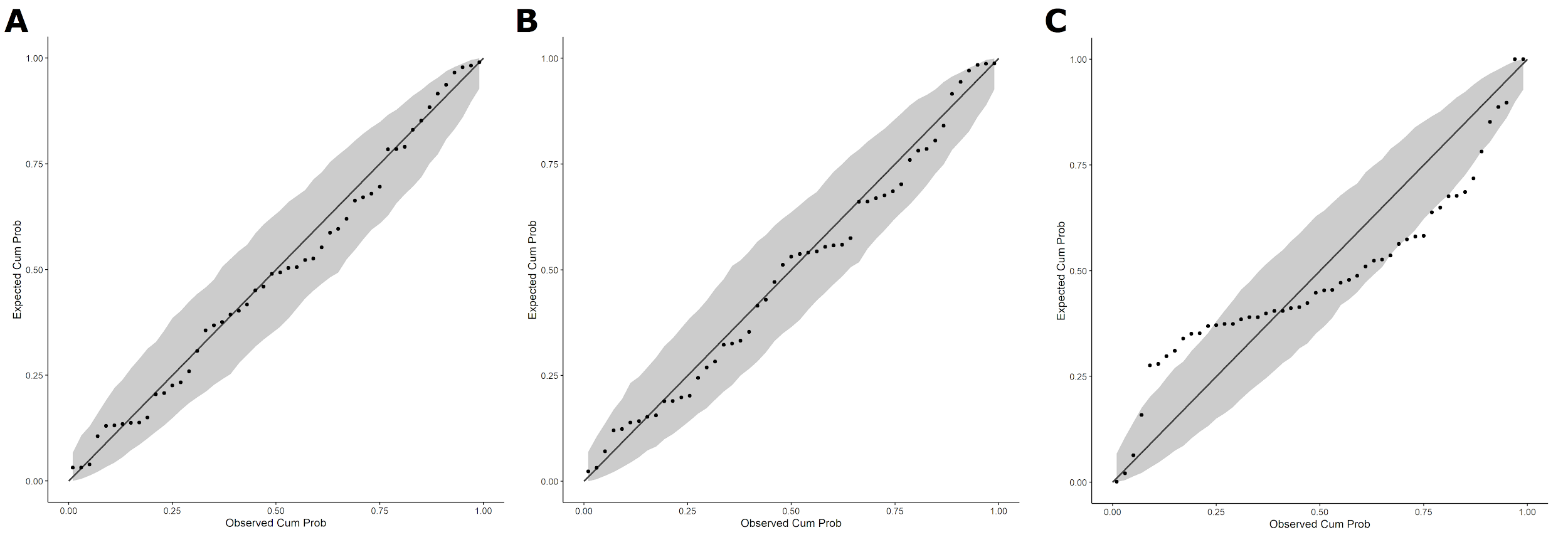


Supplementary figure 7.


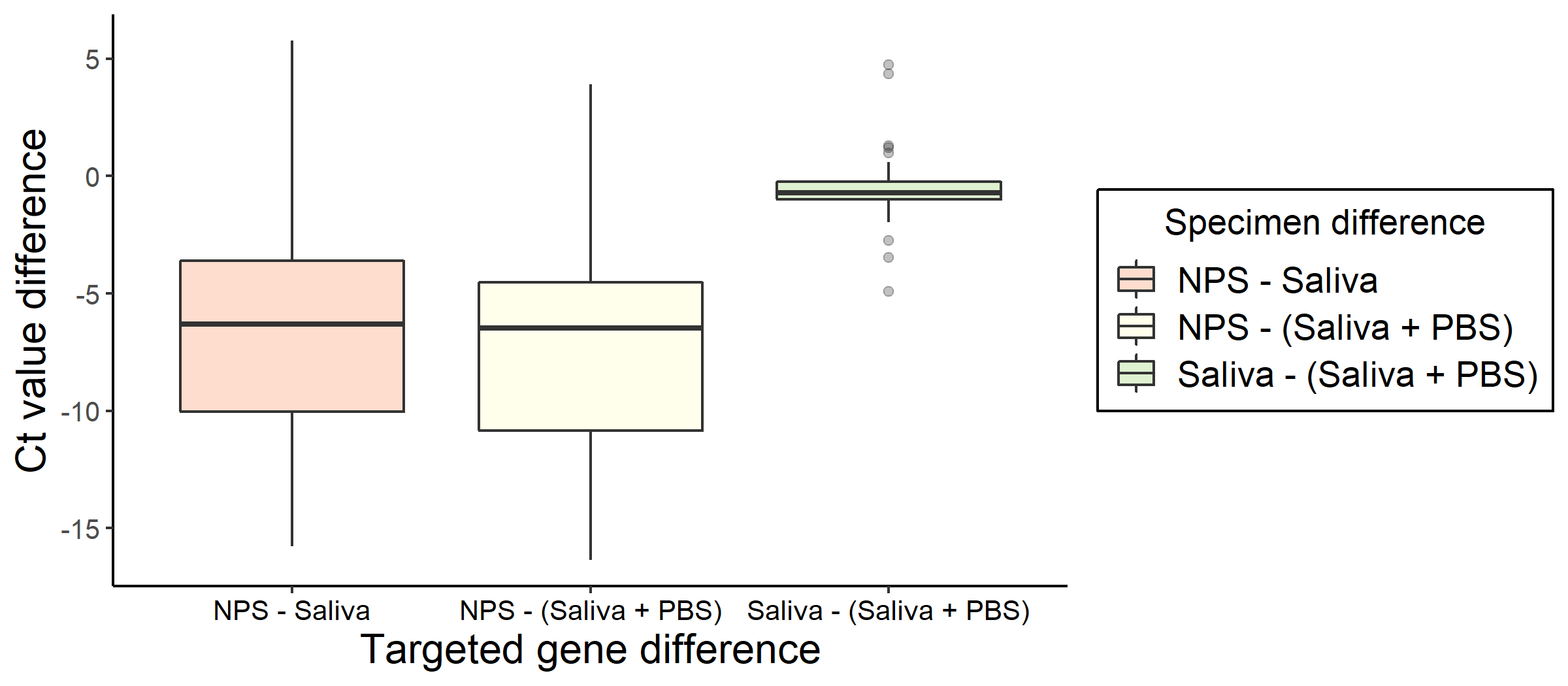


Supplementary figure 8.


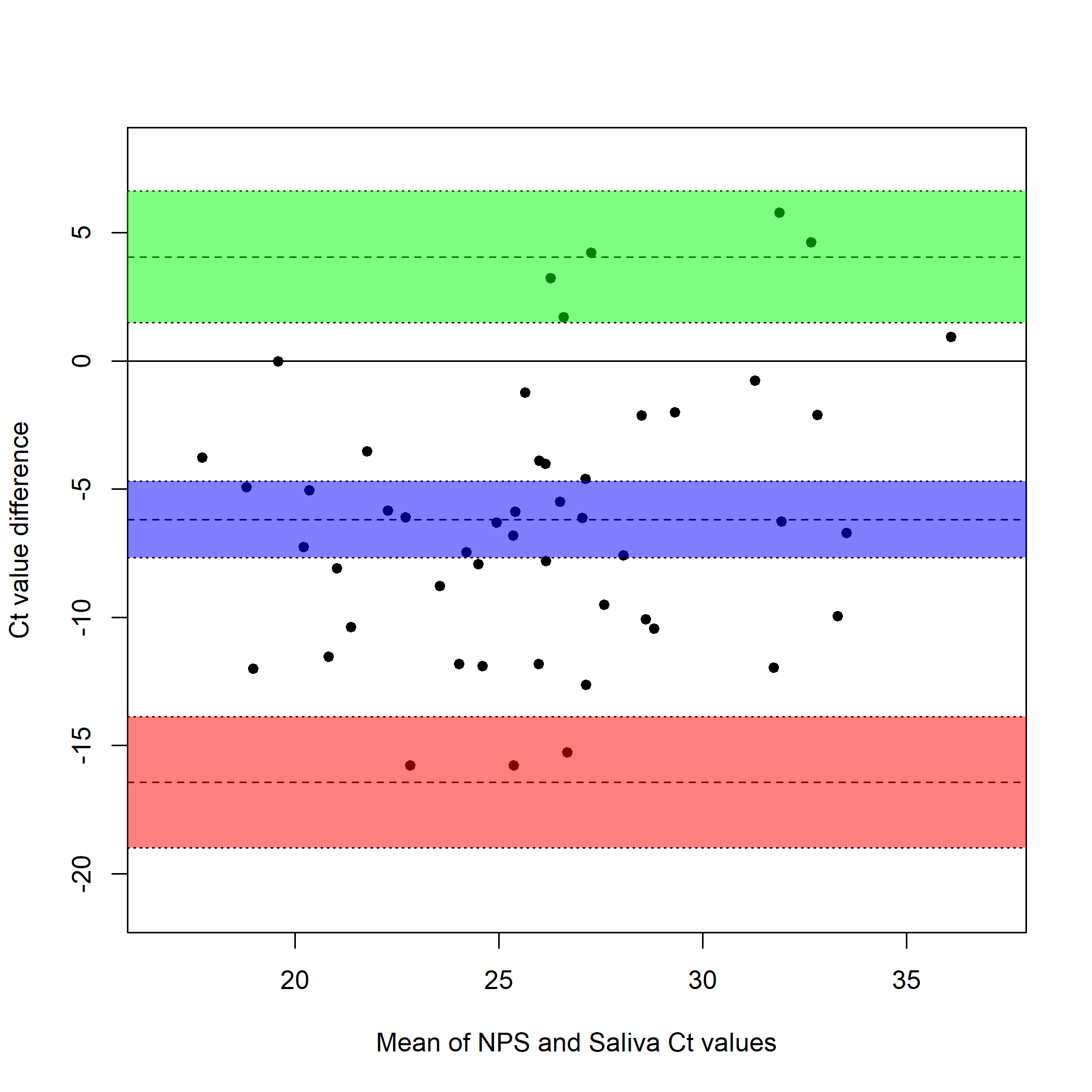


Supplementary figure 9.


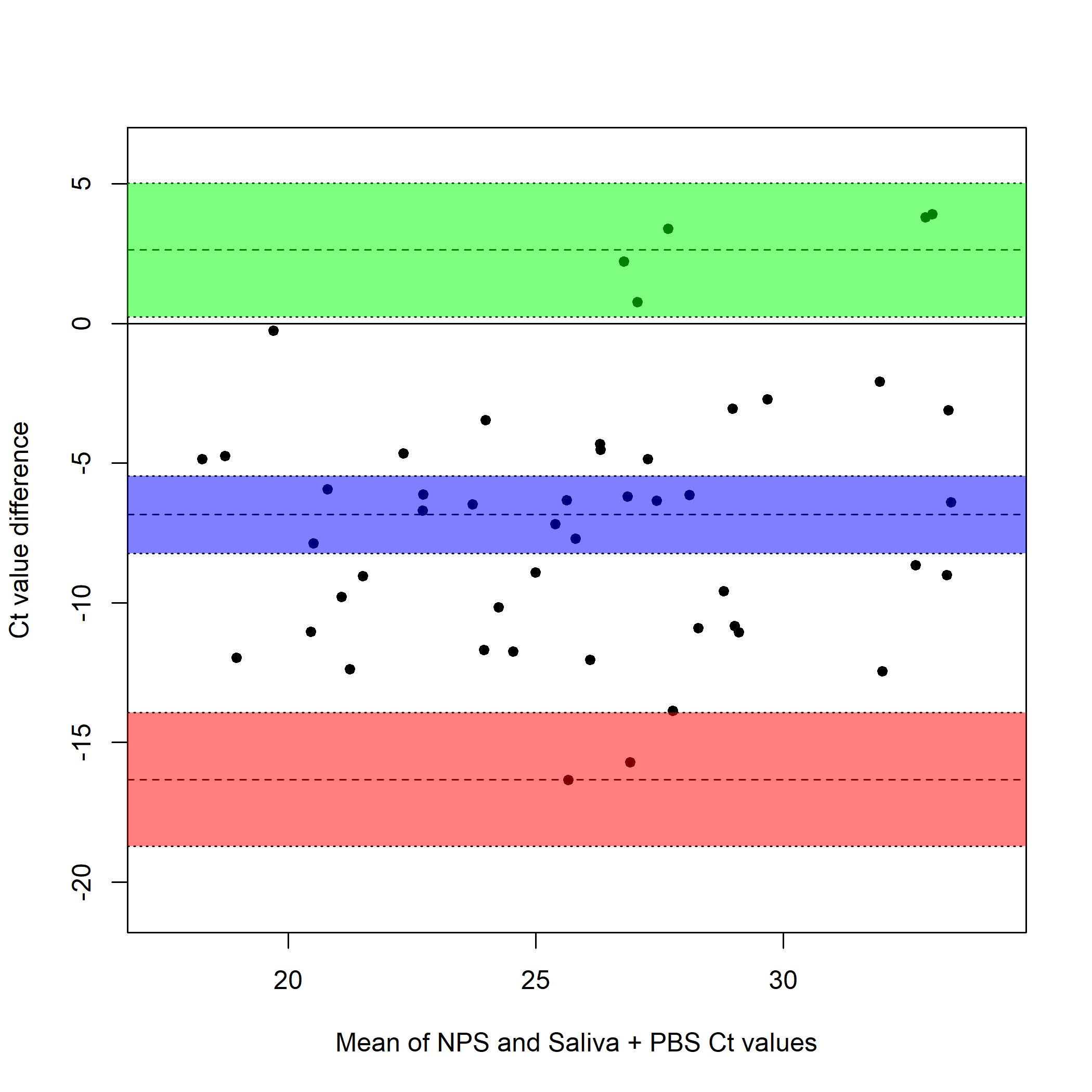

Supplement: Supplementary file 1 [file Table_1.docx]
